# Action Imagery and Observation in Neurorehabilitation for Parkinson's Disease (ACTION-PD): Development of a User-Informed Home Training Intervention to Improve Functional Hand Movements

**DOI:** 10.1155/2021/4559519

**Published:** 2021-07-23

**Authors:** Judith Bek, Paul S. Holmes, Chesney E. Craig, Zoë C. Franklin, Matthew Sullivan, Jordan Webb, Trevor J. Crawford, Stefan Vogt, Emma Gowen, Ellen Poliakoff

**Affiliations:** ^1^Division of Neuroscience and Experimental Psychology, School of Biological Sciences, Faculty of Biology Medicine and Health, Manchester Academic Health Science Centre, University of Manchester, Manchester, UK; ^2^Health, Psychology and Communities Research Centre, Department of Psychology, Manchester Metropolitan University, Manchester, UK; ^3^Research Centre for Musculoskeletal Science and Sports Medicine, Manchester Metropolitan University, Manchester, UK; ^4^School of Science and the Environment, Manchester Metropolitan University, Manchester, UK; ^5^Department of Psychology, Lancaster University, Lancaster, UK

## Abstract

**Background:**

Parkinson's disease (PD) causes difficulties with hand movements, which few studies have addressed therapeutically. Training with action observation (AO) and motor imagery (MI) improves performance in healthy individuals, particularly when the techniques are applied simultaneously (AO + MI). Both AO and MI have shown promising effects in people with PD, but previous studies have only used these separately.

**Objective:**

This article describes the development and pilot testing of an intervention combining AO + MI and physical practice to improve functional manual actions in people with PD.

**Methods:**

The home-based intervention, delivered using a tablet computer app, was iteratively designed by an interdisciplinary team, including people with PD, and further developed through focus groups and initial field testing. Preliminary data on feasibility were obtained via a six-week pilot randomised controlled trial (ISRCTN 11184024) of 10 participants with mild to moderate PD (6 intervention; 4 treatment as usual). Usage and adherence data were recorded during training, and semistructured interviews were conducted with participants. Exploratory outcome measures included dexterity and timed action performance.

**Results:**

Usage and qualitative data provided preliminary evidence of acceptability and usability. Exploratory outcomes also suggested that subjective and objective performance of manual actions should be tested in a larger trial. The importance of personalisation, choice, and motivation was highlighted, as well as the need to facilitate engagement in motor imagery.

**Conclusions:**

The results indicate that a larger RCT is warranted, and the findings also have broader relevance for the feasibility and development of AO + MI interventions for PD and other conditions.

## 1. Introduction

Beyond the more widely recognised difficulties with gait, balance, and gross motor functioning, Parkinson's disease (PD) impairs fine motor skills including hand dexterity, which are needed for the successful performance of activities of daily living [1, 2]. Sudden arrests in movement, known as “freezing,” of the upper limbs can also occur in PD, which may be correlated with freezing of gait [3]. Daily activities can be affected even in the early stages of PD [4], potentially impacting on work performance as well as household tasks, self-care, hobbies, and leisure activities. Indeed, people with PD consistently report dexterity among the domains most affected by the condition [5, 6] and have expressed a need for interventions to improve dexterity [7, 8]. However, few studies have directly addressed dexterity problems in PD [9].

Although PD affects the internal generation of action [10], external cues such as visual stimuli (e.g., floor markers) and auditory stimuli (e.g., rhythmic music) can help to elicit or control movement; this may relate to the relative preservation of goal-directed movement pathways, which compensate for impaired habitual or automatic processes [11]. However, while such cues may be effective in improving gait parameters [12, 13], they are less applicable to the fine hand movements required for everyday functional actions. Additionally, they cannot always be readily applied in real-life situations outside of the clinic or laboratory, and long-term effects of cueing have not been established [13].

An alternative type of movement cue may be provided by observation of human action (action observation, AO). A large body of the literature based on investigations in healthy participants has demonstrated that AO facilitates movement and increases motor learning [14–17]. This involves the activation of an action observation network [18], incorporating a set of frontoparietal neural structures that are engaged during both AO and motor execution, referred to as the “mirror neuron” system. Another process that shares neural substrates with AO and motor execution [19] is motor imagery (MI). MI, also referred to as *action imagery* [20], is the imagination of movement with associated sensations (kinaesthetic imagery) and images (visual imagery), in the absence of overt action [21], and is found to facilitate learning and movement in healthy participants [22, 23].

AO and MI have shown promising effects in neurorehabilitation [24–26]. In a small number of laboratory studies in people with PD, AO influenced movement speed and timing in reaching [27] and finger-tapping [28] tasks, as well as hand movement amplitude [29], and preserved motor resonance for incidentally observed hand actions has been found in PD [30]. People with PD also report similar vividness of MI to healthy controls; however, like their actual movements, their imagery may be slowed [31], and compensatory mechanisms may be involved [32, 33].

Small-scale intervention studies in PD have provided preliminary evidence that AO combined with physical practice can improve motor symptoms, balance, and gait [34, 35], as well as dexterity [36] and functional independence [37]. Increased activation in cortical motor areas has also been found following AO-based training in PD [34], suggesting potential neuroplastic effects. MI has been found to help overcome freezing of gait in people with PD [38], and MI training combined with physical practice improved timed motor performance [39].

In healthy participants, combining AO and MI has been found to produce greater behavioural and neurophysiological effects than either process in isolation [23, 40, 41], and preliminary evidence suggests that combined “AO + MI” may be effective in stroke rehabilitation [42]. However, only one study to date has investigated AO + MI in PD, which showed increased imitation of hand movements when participants engaged in MI during AO, compared to AO alone [29]. It has been proposed that combining AO and MI may increase corticospinal excitability in people with PD, thereby enhancing premovement facilitation [43]. Additionally, concurrent observation provides an ongoing visual input, which may facilitate the generation of motor imagery [40], potentially compensating for difficulties with MI that people with PD may experience [29].

To investigate the potential of combined AO + MI training to improve everyday activities in PD, we designed the ACTION-PD intervention, which utilises video-based AO + MI and physical practice of functional manual actions, delivered via an app on a tablet computer. This home-based intervention differs from previous AO therapies, which were conducted in clinics or under physiotherapist supervision (e.g., [34–36]). People with PD were involved in the development process through focus groups and as members of the research team, and our initial focus group [7] indicated that a home-based combined AO + MI intervention would be acceptable and useful, including the potential to offer personalised treatment.

Given the heterogeneous nature of PD, “personalised treatments” has been identified as a research priority by people with PD [8]. In this respect, training based on action representation (AO and MI) can be tailored to the individual's needs and rehabilitation goals. While the ultimate aim of the intervention is to develop skills in using AO + MI that individuals can apply across multiple situations, focusing on personally meaningful actions is likely to increase motivation and engagement with the training [7].

**Figure 1:**
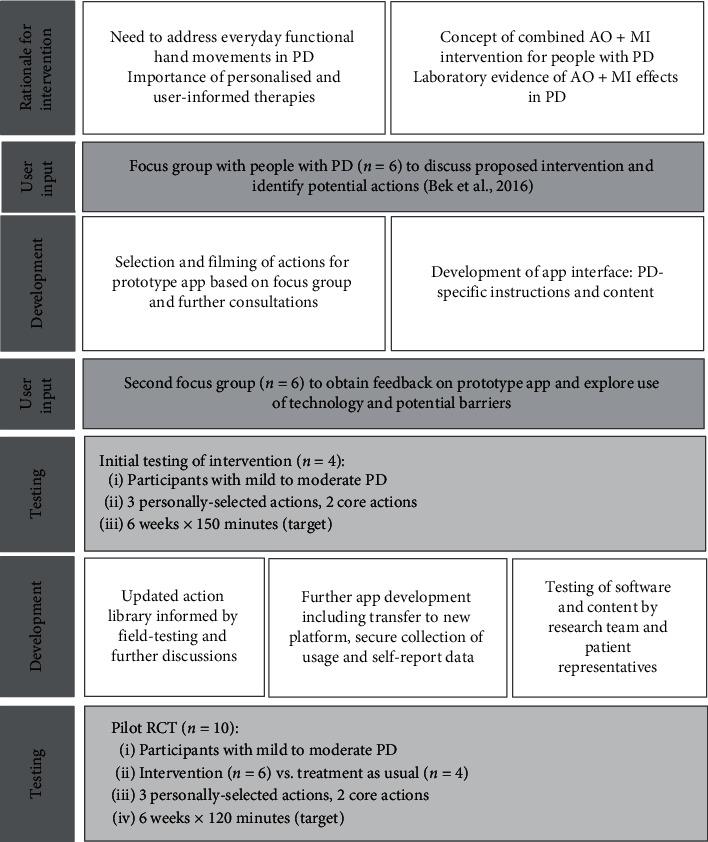
The intervention development process.

This article describes the next stages in the development and pilot testing of the intervention, which consisted of (i) design of the intervention prototype; (ii) initial field testing; and (iii) a pilot randomised controlled trial (RCT). The aim of the present study was to collect preliminary qualitative and quantitative data on usability and acceptability and to explore potential outcomes of the intervention, in order to establish whether a full RCT is warranted. The intervention development process from conceptualisation to pilot testing is outlined in Figure 1.

## 2. Methods

Ethical approval for the project was obtained from the UK National Health Service Research Ethics Committee and all participants provided written informed consent.

### 2.1. The Intervention Prototype

An action library was first compiled to enable users to select the actions they wished to train, based on suggestions from our previous focus group [7], examination of the literature, and discussions within the research team. The selection of actions was limited to those that could be practiced safely at home in a seated position, using everyday objects. Patient representatives were invited to review the library and suggest any additional actions.

**Figure 2:**
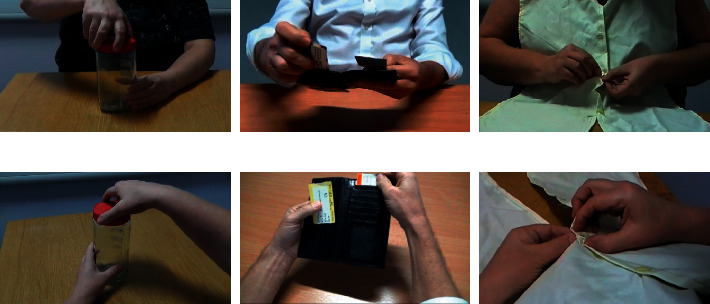
Examples of everyday actions used in the intervention (coffee jar, ticket sorting, and buttoning). Each action is presented from the third-person perspective (a) followed by the first-person perspective (b).

The actions selected to include in the prototype (see Figure 2 for examples) were video-recorded in a quiet room, using a plain wooden table and a neutral background free from other objects or distracting features. Each action was filmed with male and female actors to allow matching to the participant's gender, and from both third-person and first-person perspectives. The third-person video was filmed from either the front or side of the actor, depending on which provided the clearest view of the action, and the first-person video was filmed from the viewpoint of the actor. The third-person perspective provided the overall context of the action and movement kinematics [44], while the first-person perspective was expected to promote kinaesthetic imagery [45] and enhance sensorimotor activations [46]. Previous AO intervention studies in PD have shown positive effects using either third-person videos [28, 34, 35, 47] or first-person videos [36], suggesting that both perspectives may be beneficial.

**Figure 3:**
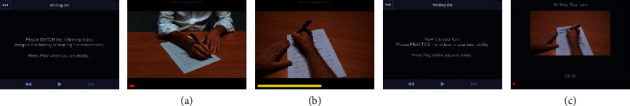
Screenshots of the prototype app used in the pilot RCT: participants were instructed to imagine each action (kinaesthetic motor imagery) while watching videos showing the action from the third-person (a) and first-person (b) perspectives, before physically performing the action using the relevant objects (e.g., pen and paper). A still image of the action (c) was displayed during action execution. Finally, participants rated the vividness of their imagery during observation and the difficulty of performing the action.

The prototype was developed through modification of an app originally designed for upper limb rehabilitation in stroke patients [48], using PD-relevant videos and updated instructions. The third-person video of the action was presented first, followed immediately by the first-person video (see Figure 3). Videos were played with the accompanying sound, which provides additional action-relevant information, and may evoke auditory activation of sensorimotor areas and facilitate motor imagery [49, 50]. Participants were instructed to observe the videos while simultaneously engaging in kinaesthetic motor imagery, which is associated with stronger sensorimotor activations than visual imagery [40]. This was followed immediately by physical execution of the action using the same objects as depicted in the video. During action execution, a still image of the action (extracted from the first-person video) was displayed on the screen as a reminder. This remained on-screen for the same duration as the preceding video, but participants were advised that they were not required to complete the action within this time limit.

A focus group was conducted with individuals with mild to moderate PD to obtain feedback on the intervention prototype and to explore views and experiences of technology more broadly (see Supplementary Materials S1).

## 3. Initial Testing and Pilot RCT

Following positive feedback from the focus group on the potential acceptability and usability of the prototype intervention, it was then pilot-tested to further explore feasibility. Exploratory pre- and postintervention measures were also collected to identify potential outcomes in terms of dexterity, reaction times, motor imagery, and quality of life. Testing was conducted in two stages: (i) initial testing with a small number of participants; (ii) pilot RCT. Below we report the methods and results of both stages together, indicating where changes were made between the initial testing and pilot RCT.

### 3.1. Participants

For the initial testing phase, four participants with mild to moderate PD and with no history of other neurological or psychiatric conditions were recruited from a volunteer panel and through Parkinson's UK (see Table 1). Participants reported experiencing difficulties with everyday manual actions, had normal or corrected-to-normal vision, and were screened for cognitive impairment [51] and anxiety and depression [52]. For the pilot RCT, a further 10 participants with mild to moderate PD were recruited and screened in the same way (Table 1).

### 3.2. Design and Protocol

#### 3.2.1. Initial Testing

With the assistance of a researcher, each participant selected 3 “personal” actions they wished to improve (e.g., buttoning, writing, opening and closing food containers). In addition, to explore the possibility of a more standardised approach to training and outcome measurement, all participants were asked to practice two “core” actions selected by the research team, which were based on common everyday tasks (handling coins, sorting train tickets). The stimulus videos (third- and first-person perspectives combined) had a mean duration of 54.9 seconds. A full list of personal and core actions is provided in Supplementary Materials S2.

Following a baseline assessment in the laboratory (see Section 3.3), a researcher visited the participant at home to deliver the tablet computer and accessory objects corresponding to the items used in the videos and to demonstrate the use of the app and explain the training protocol. A full instruction guide, as well as background information on the project and contact details for the research team, was provided within the app. Participants were also given a printed copy of the instructions. The researcher answered any questions and ensured that the participant fully understood how to use the app before independent training commenced.

The training was carried out in the individual's home for 6 weeks using the app on a tablet computer (iPad). In each training session, participants practiced the 5 actions (3 personal and 2 core), which were presented in a randomised order to avoid fatigue disproportionately affecting performance or completion of particular actions. A target training time of 150 minutes per week was set (based on previous action observation intervention studies [26]), which could be divided up according to the individual's preference. For example, if a single training session took 25 minutes, the participant could choose to complete one session per day for 6 days, or two sessions per day for 3 days. To maximise feasibility, the training was intended to be flexible, and participants were advised that they could fit their practice around other commitments or difficulties relating to symptoms.

Participants were asked to record dates and times of practice sessions in a paper-based training diary. For each session, they were also asked to rate the difficulty of performing each action on a five-point scale (very easy/quite easy/neither easy nor difficult/quite difficult/very difficult). During the training period, participants were followed up with a weekly telephone call and were also encouraged to contact the research team at any other time if they had questions or experienced technical issues.

On completion of the 6-week training period, participants returned to the laboratory for a follow-up assessment (approximately 10 weeks after baseline). Where possible, baseline and follow-up assessments were conducted at the same time of day to minimise variability in relation to medication effects and motor fluctuations. Semistructured interviews were then conducted to obtain qualitative feedback on the app and to explore individuals' experiences of the training.

#### 3.2.2. Pilot RCT

**Figure 4:**
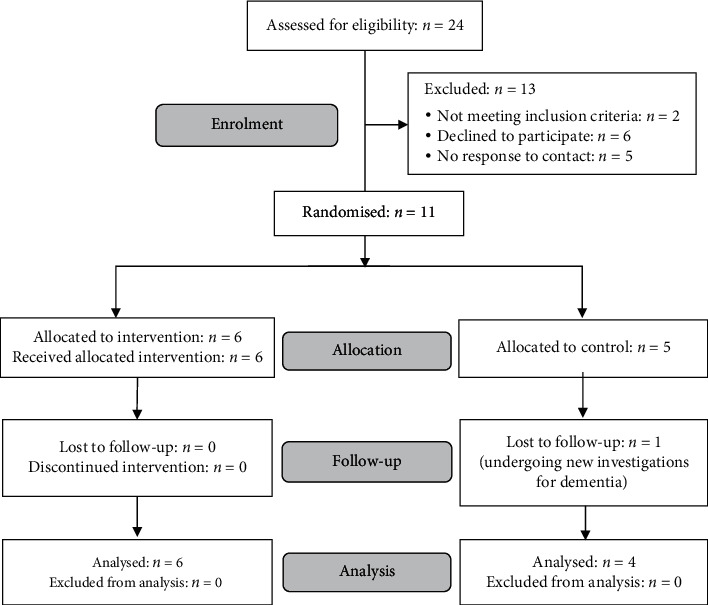
CONSORT diagram showing flow of participants in the pilot RCT.

The pilot RCT was registered with ISRCTN (trial number 11184024). The flow of participants through the trial is illustrated in a CONSORT diagram [53] in Figure 4. Prior to the pilot RCT, the app was transferred to a new software platform that enabled secure in-app collection and storage of usage and self-report data, in place of the paper-based training diaries used in the initial testing phase. A larger library of videos was also produced, based on feedback from the initial testing and further discussion within the research team. Additionally, two new “core” actions (opening and pouring from a water bottle, transferring sugar from a jar to a cup) were identified in discussion with PD representatives.

Each participant selected six actions from the updated action library in order of preference: the first three actions were included in the individual's training programme (“personal-trained”) while the other three (“personal-untrained”) were used to test for transfer of training effects. The two core actions were included in training for all participants (see Supplementary Materials S2).

Following baseline assessment, participants were randomly allocated to the intervention group or control group by a researcher who was not involved in recruitment or data collection, using an online randomisation tool.

The intervention protocol was the same as described above except for the following:

- Based on data from the initial testing suggesting that training sessions took less time than anticipated to complete and that not all participants were achieving the weekly target, the training time was reduced to 120 minutes per week. Again, this could be divided up according to the participant's preference (e.g., two 20-minute sessions per day for 3 days per week).
- Immediately after completing each action, participants completed in-app ratings of vividness for their imagery when watching the video, using a five-point scale. The difficulty of the action was then also rated on a five-point scale.

The control group participants continued with their usual treatment for PD and did not receive the intervention, but were followed up with a weekly telephone call to maintain contact.

### 3.3. Outcome Measures

Usability and acceptability were assessed through the adherence data and ratings collected via home training diaries or through the app, as described above. Feasibility was further explored through the semistructured posttraining interviews, in which participants were asked about their experiences of the app and the training content and schedule, as well as any perceived changes in their performance of the actions and transfer of skills to other tasks.

To explore potential outcomes of training, the following measures were administered before and after the intervention period:

- Dexterity for everyday tasks was assessed using the Dexterity Questionnaire (DextQ-24 [54]), a self-report questionnaire designed for people with PD.
- Quality of life was assessed using the Parkinson's Disease Questionnaire (PDQ-39 [55]).
- Motor imagery was tested using the Kinaesthetic and Visual Imagery Questionnaire (KVIQ [56]), which has been validated in people with PD [57]. The KVIQ requires participants to physically perform and then imagine performing, simple actions involving different body parts (the upper limbs, lower limbs, trunk, shoulders, and head). Visual and kinaesthetic subscales are used to rate the vividness of images and intensity of sensations, respectively.
- Simple and choice reaction time tests required participants to react to the appearance of an LED by pressing a button on a response box as quickly as possible [58]. The simple task consisted of two blocks, with responses made using the left hand in the first block and the right hand in the second block. In the choice RT task, participants responded using the hand corresponding to the location of the light signal, which appeared in a random order on either the left or right side of the display.

In the pilot RCT, performance of personalised (personal-trained and personal-untrained) and core actions was also assessed in the laboratory. Participants viewed videos showing each action from the third-person and then first-person perspective, while simultaneously engaging in kinaesthetic imagery, before physically performing the action. Each action was presented 3 times, resulting in a total of 24 trials. Videos were viewed on a projector screen (300 × 580 mm display size), approximately 1100 mm from the participant, who was seated at a table with the objects needed to complete the action placed in front of them. The objects were occluded by an opaque screen until the end of the video, when a go-signal indicated the start of the physical practice as the objects were revealed (the word “Go” in text appeared on the screen, accompanied by a beep). Following each trial, participants were asked to rate the difficulty of performing the action on a five-point scale. Action performance was filmed using a video camera positioned adjacent to the projector screen, and the time taken to complete each action was coded from the video by a researcher who was blinded to group allocation.

## 4. Results

### 4.1. Feasibility

#### 4.1.1. Training Adherence

All participants in the initial testing and those in the intervention arm of the pilot RCT completed the 6 weeks of training, with an average of 7.8 (range: 5.7–11.7) sessions per week in the initial phase and 8.9 (6–14) sessions per week in the pilot RCT. Based on an estimated average session duration of 20 minutes, this corresponds to a mean adherence of 104% in the initial cohort (76–156%) and 148.3% in the pilot RCT (99.5–233%).

#### 4.1.2. Posttraining Interviews

The semistructured interviews were audio-recorded by the researcher and transcribed by an independent transcription service. Given the overlap in content of the interviews, data from the initial testing phase and the pilot RCT were combined for analysis, which used an inductive thematic approach [59]. Themes are summarised in Table 2, and a more detailed analysis with illustrative quotes is provided in Supplementary Materials S3. Following the interview, each participant was asked to rate aspects of the app and training on five-point scales. All participants rated the app usability and the actions as either “very easy” or “quite easy” and said that they would “definitely” or “probably” use a similar app in the future. Eight of the ten participants reported that they enjoyed the training “very much” or “somewhat,” five felt that they had “definitely” or “probably” improved on the trained actions, and six reported that they had “probably” improved on other untrained actions.

### 4.2. Action Difficulty and Motor Imagery Ratings

Ratings of action difficulty and motor imagery vividness during training are summarised in Supplementary Materials S4. Across the initial testing and pilot RCT, an overall reduction in difficulty ratings between the first and sixth weeks was found for both core actions (median change = 35.1%) and personal actions (median change = 43.4%). Core actions were rated as easier than personal actions from the start of training and perceived improvements in these appeared to reach a plateau by week 2 in both cohorts. In the pilot RCT, ratings of motor imagery did not show any evidence of improvement across the 6 weeks; in fact there was a slight reduction in reported vividness (median change = 16.2%).

### 4.3. Exploratory Outcomes

**Figure 5:**
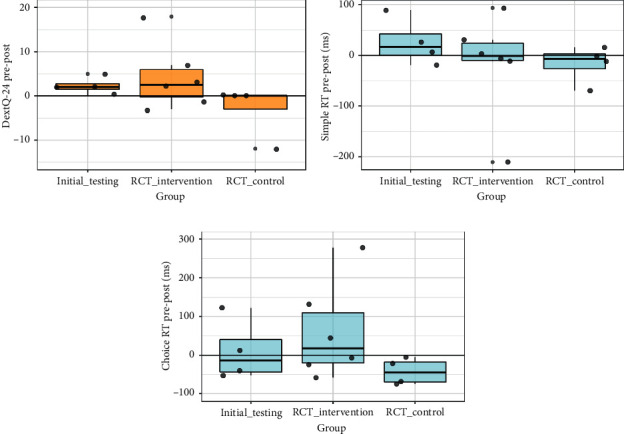
Changes in exploratory outcome measures in the initial testing and pilot RCT: (a) DextQ-24; (b) simple reaction time; (c) choice reaction time. Boxes show medians and quartiles with dots representing individual participants. Positive change indicates improvement.

Statistical analyses of the exploratory outcome measures were not performed because of the small sample sizes, but descriptive statistics are provided in Supplementary Materials S5. There was no clear indication of improvement on the PDQ-39 or KVIQ; however, numerical trends suggested the potential for improvement in self-reported dexterity as well as simple and choice reaction times (see Figure 5).

### 4.4. Motor Performance

**Figure 6:**
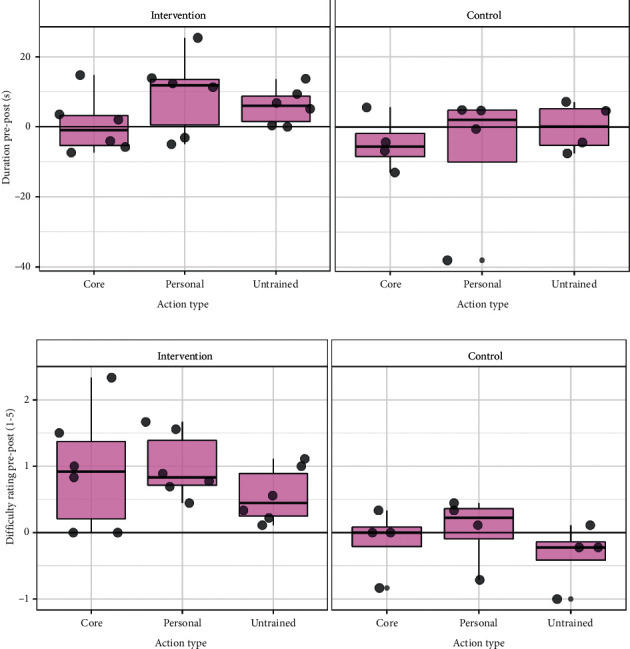
Changes in (a) timed action performance and (b) difficulty ratings in the pilot RCT for the core actions (common across participants) and personally selected trained and untrained actions. Boxes show medians and quartiles with dots representing individual participants. Positive values indicate a postintervention reduction in (a) duration or (b) difficulty.

Analysis of video-recorded action performance at baseline and postintervention in the pilot RCT indicated reduced completion times for personal-trained and personal-untrained actions and reduced difficulty ratings for all action types, in the intervention group (see Figure 6). In contrast, controls showed no indication of improvement in completion times or difficulty ratings.

## 5. Discussion

ACTION-PD is a user-informed, home-based intervention to improve everyday functional actions in people with PD through combined action observation and motor imagery. The intervention, and a prototype app for its delivery, was designed by an interdisciplinary team with input from people living with PD. Given the heterogeneity and variability of PD, personalisation and flexibility were incorporated into the intervention [7]. To obtain initial data on acceptability and usability and to explore potential outcome measures to include in a larger trial, a focus group and initial field testing were conducted, followed by a pilot randomised controlled trial. Despite some modifications to the intervention, including the implementation of a new software platform, the qualitative and quantitative findings described below were similar across both the initial testing and pilot RCT.

### 5.1. Acceptability and Usability

The focus group indicated in-principle acceptability of the app and the proposed training protocol. In both phases of pilot testing, participants were able to use the app to train independently following initial setup and guidance from the research team, as demonstrated for other home-based training programmes in PD (e.g., [60]). Initial testing indicated the need to adjust the target training dose, which was subsequently achieved by all participants in the pilot RCT.

In addition to the usage data, the posttraining interviews provided initial evidence that the ACTION-PD intervention is acceptable and usable for people with mild to moderate PD. Participants found the app and training protocol easy to use, consistent with previous reports on the feasibility of home interventions for PD using digital technologies such as exergames (e.g., [60, 61]). The flexibility of the intervention allows individuals to fit the training into their daily routine and accommodate fluctuations in levels of fatigue or other symptoms, which participants appreciated. All participants expressed an interest in using a similar app in the future and felt that the six-week duration of the current intervention was appropriate. While some participants found the actions well-suited to their needs, not all of the actions were considered to be sufficiently challenging. Indeed, it was suggested that the possibility of selecting new actions, or progressing to more challenging actions, could make training more motivating and sustainable. The focus group and posttraining interviews also highlighted the value of feedback and encouragement to maintain motivation, consistent with previous findings in relation to other interventions for PD [7, 58, 63].

Subjective ability to perform the motor imagery component of the intervention varied between participants. Some individuals found it difficult to engage initially but easier as training progressed, while others felt that their imagery did not improve over time. In this context, it should be noted that motor imagery ability varies widely among the general population [64], and although vividness of imagery is generally found to be preserved in PD, it may be affected in some cases [31].

Participants generally reported a preference for observing actions from the first-person perspective, although the overall contextual information provided by the third-person viewpoint was also appreciated (see Ewan et al. [44] for similar findings in stroke survivors). Evidence from spontaneous gestures when describing actions suggests that people with PD may rely more on the third-person perspective to internally represent movement [65, 66]; nonetheless, the preference for the first-person video suggests that observation from this perspective may facilitate the generation of first-person kinaesthetic imagery by providing a visual prompt, as highlighted in the following quote: “I'd feel more what that felt like to me, because the film was about…as if it was me that was doing the action.” This is consistent with the hypothesised role of AO within AO + MI as providing an external visual guide for MI, as indicated by MI-specific effects on corticospinal excitability in healthy participants [67].

### 5.2. Potential Outcomes of AO + MI Training in PD

Posttraining interviews identified perceived improvements in performance of the trained actions, as well as other daily activities, indicating the potential to achieve broader benefits beyond task-specific training effects. However, some participants reported that improvements occurred early into the training period, with limited further progress, again highlighting the importance of progressive training.

Several participants reported using MI in everyday tasks following the training, such as when dressing or getting out of bed. Additionally, the interviews indicated other ways in which AO + MI training may have influenced how participants approached actions. These included (i) focusing attention so that tasks could be carried out in a more careful and controlled manner, as recommended in physiotherapy guidelines [68] and which speculatively could be linked to increased use of MI; (ii) reducing the stress associated with performing difficult actions; or (iii) highlighting subtleties of the movements. Potential psychological benefits including increased confidence and self-efficacy were also noted, consistent with other literature studies reporting these functions of motor imagery in older adults [69].

Analysis of action performance in the pilot RCT showed that completion times for both trained and untrained personally selected actions were shorter following training in the intervention group, which corresponded to decreased difficulty ratings in the laboratory. This was broadly consistent with the pattern of difficulty ratings collected during training, which indicated that participants generally found the practiced actions easier by the end of the six-week period. However, most found the “core” actions selected by the research team less challenging than the “personal” actions that they had selected themselves, reinforcing the importance of personalisation.

Numerical trends in the exploratory outcome measures also suggested the potential of AO + MI training to improve dexterity and reaction times, which requires further investigation in a larger trial. A self-report dexterity measure was used in the present study because of its direct relevance to the everyday actions targeted, but in future studies this should be complemented by objective tools [70]. A large trial of home-based training with task-specific hand exercises compared to resistance training in people with PD found improved performance on a peg test alongside self-reported dexterity [60], and the only previous study to investigate effects of AO training on dexterity in people with PD also found improved performance on a peg test [36].

Consistent with the findings from the interviews discussed above, in-app ratings of motor imagery in the pilot RCT did not show any subjective improvement across the six weeks of training, and there was no clear indication of improvement in motor imagery ability on the KVIQ. However, such self-report measures rely on the individual's understanding of the concepts in question, and obtaining reliable longitudinal data requires consistent interpretation of the instructions over time. Indeed, some participants in the present study showed an altered understanding of imagery as a result of the training. Additional instruction and training in MI prior to the intervention might therefore improve understanding [69] and engagement, as well as consistency of both the measurement of imagery and its use within the intervention. Future work should also consider how best to evaluate changes in the everyday use of MI in people with PD, as indicated by the reports of some participants in the present study, which may not be adequately captured by commonly used tools assessing imagery vividness.

### 5.3. Proposed Mechanisms and Future Work

These preliminary findings demonstrate the potential for combined AO + MI training to facilitate everyday functional manual actions in people with PD. There are several mechanisms by which this may be achieved. First, specific motor representations for the trained actions may be developed or enhanced through AO and MI alongside physical practice [71, 72]. Second, the training may result in improved ability to generate MI for the practiced actions, such that participants are able to apply imagery more easily when performing the same actions outside of the training context. A third possibility is that participants develop stronger general skills in MI or a greater awareness of MI, which they are able to apply to functional actions beyond those practiced, as suggested by the perceived improvement in performance of untrained actions in the pilot RCT. Finally, as suggested by the qualitative findings, AO + MI training may lead to other changes in how actions are approached, such as focusing attention [73] or increasing confidence and self-efficacy [69]. Indeed, the training may produce a combination of these outcomes. Cognitive-motor and psychological mechanisms such as those above would indicate effects beyond physical practice alone and should be further explored in future research.

Individual differences (for example, in motor imagery) may also influence the efficacy of home-based AO + MI training, such that some participants may obtain greater motor, cognitive, or psychological benefits than others. In future, it may be appropriate to screen individuals to ensure a minimum level of MI ability prior to training, as in some previous studies of interventions for stroke [74]. Additionally, the qualitative data suggested that motivational factors vary between individuals, with some finding intrinsic motivation from the daily routine or the potential to improve their movements, while others may rely more on extrinsic motivators.

Themes relating to personalisation, variety and choice, and motivation were revealed by the posttraining interviews, which also echoed the findings of the focus group (Supplementary Materials S1). In summary, key issues highlighted for further development of the intervention include (i) selecting appropriate actions at a suitable level of difficulty for the individual; (ii) offering variety, choice, and progression in training; (iii) providing additional guidance or instruction to facilitate engagement in motor imagery; and (iv) increasing or maintaining motivation, through the above, as well as via positive reinforcement and feedback.

The present findings indicate that home-based AO + MI training delivered using mobile technology is feasible in people with mild to moderate PD, although future work should explore the feasibility of the intervention in those with more severe symptoms or in different subtypes. Home-based approaches could provide widely accessible, low-cost, and scalable alternatives or supplements to existing rehabilitation programmes, and their importance is more apparent than ever in light of the COVID-19 pandemic [75, 76].

Based on the findings of this pilot work, the intervention should be evaluated in a larger-scale randomised controlled trial, following further development with input from people with PD and healthcare professionals. Additionally, the involvement of healthcare professionals in prescribing appropriate training content and setting up the intervention should be considered. The findings also have broader relevance for the development of behavioural interventions in PD, as well as applications of AO + MI in other groups, such as stroke survivors or healthy older adults.

## Figures and Tables

**Table 1:** Baseline characteristics of participants in pilot testing.

Participant	Sex	Age (years)	Time since diagnosis (years)	Hoehn and Yahr stage	UPDRS-III motor score
Initial_1	M	73	7	2	54
Initial_2	F	72	10	3	36
Initial_3	M	63	8	1	16
Initial_4	F	50	2	2	32
RCT_I1	M	70	4	2	49
RCT_I2	M	65	7	2	29
RCT_I3	M	71	4	2	40
RCT_I4	M	66	16	2	37
RCT_I5	F	69	2	3	47
RCT_I6	M	60	2	3	66
RCT_C1	M	66	13	2	51
RCT_C2	M	59	5	2	39
RCT_C3	M	63	2	1	28
RCT_C4	M	47	4	2	42

*Note*. Initial = initial testing cohort; RCT_I = pilot RCT intervention group; RCT_C = pilot RCT control group.

**Table 2:** Themes generated from semistructured posttraining interviews.

*Theme 1: suitability and choice of actions*
The interviews revealed mixed experiences of the actions practiced within the training. Several participants reported that the actions were unchallenging or that they found only one or two of the actions difficult. Other participants found the actions well-suited to their needs or appreciated the combination of easier and more difficult actions. Some participants noted that it was useful to practice everyday actions that would be commonly encountered. On the other hand, the disparity between practicing the actions at home and in real-life scenarios was discussed.
All participants felt that the intervention would benefit from a greater variety and choice of actions. It was suggested that individuals could be supported to select actions appropriate to them. Some participants would like the option to replace actions once a level of competence had been achieved or to be able to progress to more difficult actions. One participant felt that they would prefer to focus on one action at a time, according to their current needs.

*Theme 2: action observation and motor imagery*
It was noted that watching the videos provided useful cues for improving performance, and one participant reported that this was particularly helpful for the more difficult actions. It was also suggested that watching the videos could increase awareness of variability in the observer's own actions. However, one participant noted that they became distracted while watching the videos, so they may not have always fully attended to the presented action.
Participants generally reported a preference for viewing actions from the first-person perspective, which for some individuals could change over time. Comments indicated that the first-person video promoted motor imagery, although some participants appreciated seeing the third-person view first to obtain an overall understanding of the action. Some participants felt that it was helpful to see both perspectives, which might facilitate motor imagery and learning.
Individual differences in experiences of the motor imagery component of the training were highlighted. Some participants found it effortful to engage in motor imagery, which either improved over time or remained problematic, while other comments indicated that the importance of the imagery component might be unclear. Hearing the sounds associated with the actions was suggested to help in facilitating imagery.

*Theme 3: accommodating the training within everyday life with Parkinson's*
Participants generally found the training schedule manageable and were able to fit the session into their day, valuing the flexibility to work around other commitments and activities. However, one individual commented on the additional time needed to set up the objects in preparation for their session, which increased the daily time demands. Another person found that their sessions took quite some time to complete and that they had sacrificed other activities in order to fit in the training. The duration of the current intervention period was generally found to be acceptable and appropriate.
Some participants noticed that their ability to perform the actions was impacted by medication effects or fatigue, which could result in inconsistent performance at different times of the day. The variable nature of Parkinson's, including fluctuation of symptoms and the way the condition could affect different actions, was also commented on by several participants.

*Theme 4: perceived effects including cognitive and psychological changes*
Most participants noticed at least some degree of improvement in the actions trained within the intervention, although others did not perceive any change in their performance, which in some cases was suggested to relate to the suitability of the selected actions. The training had helped some participants in performing other everyday tasks. Comments suggested that this could relate to a change in attitude or mindset when approaching actions.
Some participants more explicitly referred to changes in awareness or use of action representation processes (observation and imagery) in everyday life, although some did not notice any such changes. Examples of applying imagery to specific tasks were provided, including tool use, dressing, getting out of bed, and moving through doorways.
Other changes such as increased confidence, sense of control, and self-efficacy were reported by some participants.

*Theme 5: the importance of motivation and feedback*
Motivation was unanimously considered an important issue in home-based training, although individuals' views on what would motivate them differed.
For some participants, the potential to improve movements through the training, or just the achievement associated with completing the daily sessions, was intrinsically motivating. Practicing more challenging actions, or a progression in the difficulty of actions, might also provide a source of motivation.
External sources of motivation were also highlighted. Some participants said that they would find performance-related feedback helpful. It was also suggested that more feedback and encouragement could be built into the app.

## Data Availability

The anonymized data used to support the findings of this study are available from the corresponding author upon request.

## Supplementary Materials

Supplementary Materials The following supplementary files are available: S1: focus group. S2: everyday actions included in pilot testing. S3: themes from posttraining interviews. S4: difficulty ratings and motor imagery ratings during home training. S5: exploratory outcome measures.
