# Supplementary material for: Action Imagery and Observation in Neurorehabilitation for Parkinson's Disease (ACTION-PD): Development of a User-Informed Home Training Intervention to Improve Functional Hand Movements
Source: Parkinsons Dis. 2021 Jul 23;2021:4559519. doi: 10.1155/2021/4559519 (PMC8324342; doi:10.1155/2021/4559519)
Supplement: Supplementary Materials — The following supplementary files are available: S1: focus group. S2: everyday actions included in pilot testing. S3: themes from posttraining interviews. S4: difficulty ratings and motor imagery ratings during home training. S5: exploratory outcome measures. [file 4559519.f1.pdf]

Supplement S1: Focus group

A focus group was conducted with 6 participants (4 males, 2 females) with mild to moderate PD (Hoehn & Yahr stage 1 to 3) who were between 3 and 21 years post-diagnosis. Five of the participants had attended our previous focus group[7] and one was a member of the research team; all participants were therefore already familiar with the concepts of AO and MI and the principles underlying the intervention.

The focus group was chaired by one of the authors (EP) and facilitated by two others (JB and JW). A schedule of topics was used to guide discussions, with open-ended questions and additional prompts where needed. The prototype app was demonstrated and participants were then invited to try the app themselves and comment on the interface and functionality. The proposed training protocol was also discussed, as well as the use of technology more broadly, including participants' experience in using mobile devices and apps, and potential symptomatic barriers to technology use. The following themes were identified through inductive thematic analysis.

Responses were recorded and transcribed by an independent transcription service. Using thematic analysis with an inductive approach, themes relating to (i) accessibility, (ii) motivation, and (iii) personalisation and flexibility, were identified (Table 1). Further details of the focus group and a full list of themes with illustrative quotes are provided in the supplementary material (S1).

Theme 1: Accessibility of technology

Comments highlighted the need for application compatibility across a range of devices and operating systems, to ensure that the training is as widely available and accessible for people with PD as possible:

> *…I have a Windows phone, nobody makes apps for them. [FG2]*

Participants discussed how symptoms of PD could potentially interfere with the operation of the app, and that a simple interface would be appreciated:

> *I use an iPhone a lot, and fortunately, I've got very little tremor as yet, but I miss the keys, I misjudge it, very, very often, and I get the adjacent key to the one I want, and that's frustrating. [FG3]*

Theme 2: Motivating influences: progression, encouragement and feedback

It was noted that having a greater variety of actions to choose from would help to maintain motivation. In particular, the ability to progress from simpler actions to more complex sequences was suggested, and a simple game-like reward system could be incorporated:

> *I think to me, it's…about having progress. So that you get, you know, this is level one, and then you can always progress to level 2, if you've mastered that, as it were. [FG5]*

It was also acknowledged that rewards may be realised after longer-term use of the app, in that training may help to maintain the ability to perform actions that otherwise may diminish with disease progression:

> *With Parkinson's, you're actually working against the tide. So, in many ways, you need to be doing things before you lose the skills. It's about conserving, not just learning, isn't it. [FG5]*

The importance of positive reinforcement or encouragement was noted, which could take the form of either performance-based feedback or more simply recognition of regular practice.

> *We talked about the competitive element, didn't we, about knowing how well you were doing previously. We said that we'd like to know how we're doing. [FG4]*

> *Is there any sort of built in encouragement in, saying, particularly for people who do it every day, or, saying, oh great, you've done it, that's two days on the run. [FG6]*

Theme 3: Personalisation and flexibility in training

Participants discussed the importance of flexibility in the training protocol, such as in relation to the timing of medication, or to minimise fatigue effects:

> *A crucial feature would be the timing in relation to the last dose of tablets. [FG3]*

> *I'd like, for me, the idea of more small sections, more short sections, rather than a half hour block. Because it feels preferable to me, I could do a half hour block, but I think I'd start to be wandering by the end of it. [FG2]*

It was also noted that personal preferences might differ in terms of the device used to deliver the training:

> *I think the more flexible… it is, the better, because people will have different preferences. And because it's all about motivation, actually. [FG5]*

There was some discussion of features that could be incorporated within the app in future, such as reminders or music. It was acknowledged that these might be appreciated by some users but not others, and it was suggested that these elements could be optional:

> *You could have it like a permissive thing, if you could switch on a reminder. So if somebody who valued their reminder could do it, but it wasn't mandatory [FG5]*

> *I would want to be discreet, and not draw attention to the fact that I'm doing my exercises, so I don't want music playing [FG1]*

Supplement S2: Everyday actions included in pilot testing

Action name and description	Video duration (mm:ss)	Participants
Core actions		
Ticket sorting: picking up a wallet from the table; opening the clasp; selecting and removing a train ticket; fastening the wallet; placing the ticket and wallet down on the table	00:40	All Initial
Coin handling: picking out two coins from a selection held in the palm of the non-dominant hand; transferring the coins to the palm of the dominant hand	00:23	All Initial
Water bottle: unscrewing and removing the lid of a small water bottle; pouring water into a cup; replacing the lid; lifting the cup towards the mouth	00:56	All RCT
Sugar jar: unscrewing and removing the lid of a jar, extracting a teaspoon of sugar; depositing the sugar into a teacup; placing the spoon down; replacing the lid	00:49	All RCT
Personal actions		
Buttoning: fastening and then unfastening 3 buttons on a tabard	01:01	All Initial; RCT3, RCT5
Food cutting: using a knife and fork to cut 3 slices from a block of modelling clay shaped into a 'steak'	00:50	I1, I2
Butter container: removing the lid of a plastic butter container	00:16	I2
Breakfast cereal: opening a cereal box; pouring cereal flakes into a bowl; re-closing the box	00:34	I3
Lock and key: inserting a key into a padlock; turning the key to unlock the padlock; *removing the key*	00:34	I4, RCT5
Coffee jar: unscrewing the lid of a large coffee jar; screwing the lid back on	00:48	I4
Zipping: fastening and then unfastening a zip on a jacket	00:41	RCT1, RCT5
Writing - patterns: using a pen to draw pre-writing patterns on lined paper; maintaining the amplitude of the letters between the lines; completing 3 rows of different patterns	00:52	I1, I3
Writing- letters: using a pen to write letters of the alphabet on lined paper; maintaining the amplitude of the letters between the lines; completing 3 rows of different patterns	03:32	RCT2, RCT3

Action name and description	Video duration (mm:ss)	Participants
Newspaper: turning two pages or a newspaper; turning back to the cover	00:45	RCT1, RCT2
Shirt sleeves: fastening 3 buttons on a shirt sleeve cuff; repeating on other sleeve	00:54	RCT2, RCT3, RCT4
Ticket sorting: picking up a wallet from the table; opening the clasp; selecting and removing a train ticket; fastening the wallet; placing the ticket and wallet down on the table	00:48	RCT6
Ticket removing: holding a wallet in one hand and set of tickets in the other; selecting a ticket; opening the wallet; placing the other tickets in the wallet; closing the wallet while holding the selected ticket	00:44	RCT6
Paper tidying: gathering loose papers into a pile; picking up and straightening the papers; placing the pile back onto the table; picking up one sheet from the top	00:48	RCT6
Coin jar: unscrewing the lid of a small jar and placing it onto the table; picking up 4 individual coins and dropping them into the jar; screwing the lid back on	01:03	RCT4
Yogurt pot: removing the lid of a yogurt pot and placing it on the table; placing the lid back on	00:37	RCT1
Food bag: opening a zip-lock food bag; removing a cookie and placing it onto a plate; re-sealing the bag	00:44	RCT4

Note: I = initial testing cohort; RCT = pilot RCT intervention group.

Supplement S3: Themes from post-training interviews

Theme 1: Suitability and choice of actions

Some participants noted that it was useful to practice everyday actions that would be commonly encountered. On the other hand, the similarity of the actions to the 'real-life' versions was questioned.

> *Well, I thought, they were probably spot on really cause they, sort of, hit the problems that people do have, you know, the fastening of buttons and zips and very common place problems [I5]*
>
> *…the worst is if I'm out and about and there's more pressure on me and I'm there in front of the ticket collector and he's got a big queue gathering behind me and I'm … slow, and that's where things really go pear shaped [I6]*

Several participants reported that the actions were unchallenging, or that they found only one or two of the actions difficult. Other participants found the actions better suited to their needs, or appreciated the combination of easier and more difficult actions.

> *I think I regretted that I hadn't chosen some of the other actions on the list that was given to me, which might have been a bit more challenging so that I could have practiced those and maybe noticed an improvement in those or not, as the case may be, and I think I picked ones that were too easy really [I2]*
>
> *I found some easier than others but that didn't really matter and, in a way, I used to sort of shuffle the order of what I did quite a lot, the easier tasks almost gave me a bit of a pause [I6]*

Nonetheless, all participants felt that the intervention would benefit from having a greater variety and choice of actions, and it was suggested that participants could be supported to select actions appropriate to them. Participants would like to have the option to replace actions once a level of competence had been achieved, or to be able to progress to more difficult actions.

> *You or the person setting up the app, would probably need to, somehow identify with my help, which actions I can really benefit from, before embarking on the six-week test……make them progressively more difficult, maybe. You could have the writing going on to the end of the alphabet or extend it in some way [I2]*
>
> *It needs to have a larger variety of different activities, videos...You need like a menu of all the different actions and then you just pick out half a dozen. Then, when you feel you've mastered those, you can pick out some more. (P3)*

One participant felt that they would prefer to focus on one action at a time, according to their current needs.

> *I might have 30 different actions on the thing and I'd think, hmm, this is one I've been struggling with recently, and I'd just work on that one for two weeks. And then, yeah, I could select another if I needed it and work through that and then maybe check back every so often to see if the skills are still there [I6]*

Theme 2: Action observation and motor imagery

It was noted that watching the videos provided useful cues for improving performance, and one participant noted that the videos were particularly helpful for the more difficult actions. It was also suggested that watching the videos could increase awareness of variability in the observer's own actions. However, one participant noted that they became distracted while watching the videos.

> *…the films help you get into the right mind-set as well as watching again how to do it well or just how to do it in an easier manner…I think what helped was doing it so often you noticed little things which could make a difference potentially. (P4)*

> *…watching the way the she did it and the way I did it, I know that the way I did it varied a lot. (P2)*

Participants generally reported a preference for the first-person perspective, but this could change over time. Comments indicated that the first-person video may have promoted motor imagery, although some participants appreciated seeing the third-person view first to get an overall understanding of the action. Some participants felt that it was helpful to see both perspectives, which might facilitate motor imagery and learning.

> *When I first saw them, the first one, it's like the third person. I thought that was fine. Then, when I saw it in the first person, I thought that's better. But, after a while, I just thought, no, there's no difference between them really. (P3)*

> *the views of the first person they were more helpful than the view facing the person but maybe that was necessary to give context to the exercise anyway [I6]*

> *It was useful to have the two different perspectives and that helped with the visualising of the thing. [I2]*

> *To help you re-learn tasks, having it from the two angles would be better. [I3]*

Participants reported mixed experiences of the motor imagery component of the training, but this may have improved over time.

> *At first, I was really concentrating on what had to be done but as time went on, as it became easier, there was less thought going into it…it became a natural part of it [I4]*

Some participants found it effortful to engage in motor imagery, while other comments indicated that the importance of the imagery component might be unclear.

> *I put it down to practice and really trying, trying very hard. Sometimes, just closing my eyes and trying to imagine the action or the feeling. (P3)*

> *I'm not sure how much I was really accessing the imagery or whether I was just really playing the game as it were, really. It was hard to say how much I was really deriving from this concept imagery [I2]*

Hearing the sounds associated with the actions may have helped to facilitate imagery:

> *…sound effects were definitely helpful, I think without the sound there was a bit more distance between myself and the image, with the sound it was more as though I was there [I6]*

<u>Theme 3: Accommodating the training within everyday life with Parkinson's</u>

Subtheme 3.1. Acceptability of time demands

Participants generally found the training schedule manageable, and felt that they were able to fit the sessions into their day, valuing the flexibility of the intervention.

> *I think in terms of the number of exercises, that was about right. If you made it six or seven a session, I think that would be too much….at times, it was quite hard to fit in one in a day. But then there were other…I mean, I did two in a day in some cases. (P2)*
>
> *I liked the fact that you could do it whenever you had time to do it. For me on some weeks that was at different times of the day, and I think if it had been rigid it would have been more difficult to fit it in (P4)*

However, one participant commented on the additional time needed to set up the objects in preparation for their session:

> *Even though it only takes about 15 minutes, all your preparation and then putting it all there afterwards and that, takes up a good half hour, I found, really. …. when you're busy, you've got to find the space and the time to do it in… I enjoyed doing it, but I didn't enjoy doing the preparation for it. (P3)*

Another found the schedule more demanding and found that they had sacrificed other activities in order to fit in their training:

> *It did take quite a chunk out of the day, to get it in I dropped off the physio exercises which probably...I mean I do those about three times a week, that's probably not a good thing to drop off [I6]*

The duration of the current intervention was found to be manageable and appropriate:

> *I think six weeks was enough, otherwise I think it would get too, how can I put it? A chore, rather than…I don't know what word to use, yes, quite a chore rather than…you wouldn't enjoy it so much [I4]*

Subtheme 3.2. The impact of medications or symptoms on training.

Some participants noticed that their performance was affected by medication effects or fatigue.

> *And I tried doing those when I was…like at different times and when I knew I was short of medication, and that did take a long time. (P2)*
>
> *my hand movements suffer when I'm not fully charged with medication and I did find that the actions that I had to do were sometimes a bit more difficult when I was off my medication [I2]*
>
> *I found that if I left it late in the day, it was more tiring than doing it in the morning, as in my energy levels would run out for the day [I3]*

The variable nature of Parkinson's and the way it can affect different actions was also commented on by several participants:

> *I did find it more difficult if I'd had dyskinesia, it was very difficult to do things like the zip …if you're having a bad day, you might have done very well the day before, say, on the zips and the next day you might be absolutely rubbish, so you don't get a consistency, I don't think, you get good days and bad days [I5]*

<u>Theme 4: Perceived effects including cognitive and psychological changes</u>

Subtheme 4.1: Performance, mindset and imagery.

Most participants noticed at least some improvement on the actions trained within the intervention, although some did not experience any improvement, which possibly related to the suitability of the selected actions.

> *From my point of view that there's not much doubt it had a beneficial effect [I6]*
>
> *I felt they're split almost into two categories, I had two that I found very straightforward and simple and I had three that were quite difficult. They never seemed…you know, none of those really ever changed at all (P1)*

The training had helped some participants in performing other everyday tasks. Comments suggested a change in approach or mindset when performing actions:

> *It made me a bit more attentive about the everyday things, and I wasn't expecting to have that benefit…Even things like I struggle changing gear and I just thought well, maybe you're putting your hand in the wrong position, maybe in a different position it might be helpful…opening a bottle of milk or opening the door or any of those things that the actions related to it made things a bit more helpful in everyday life. (P4)*
>
> *…putting the mind into action before I do the task …it doesn't take that long to…it's not a deep thought, it's just a process of what I've got to do [I4]*

Some comments more explicitly referred to changes in awareness or use of action representation processes (observation and imagery) in everyday life, although some participants did not notice any such changes. Specific examples included applying imagery to tasks including tool use, dressing, getting out of bed and moving through doorways.

> *The idea of imagery hadn't entered my knowledge, sort of thing. And with the way of imagining things happening, I think that's given me an insight into other things, so as a spin-off from it…I think possibly subconsciously you're analysing what you're doing in all the tasks as if you've been watching a video [I3]*
>
> *when I'm getting dressed one thing I always find quite difficult is putting a leg into trousers... I try to imagine what I'm doing and then remove the leg before I tried to actually do the full action [I1]*

Subtheme 4.2: Psychological effects

Other changes such as increased confidence and sense of control were reported by some participants.

> *…doing this helped me realise that there's some things I could work on and potentially then control sometimes how I do something. So like a simple action like turning the coffee jar, originally I thought I just can't do that anymore, or it's just difficult to do it, whereas practicing it made me think actually you can control things a little bit more than you think you can and not accept that you think you can't do something (P4)*
>
> *I found that practicing took away an element of the stress and made the whole thing a bit easier and a bit simpler to do. So, I found it quite helpful, in that regard [I2]*

Theme 5: The importance of motivation and feedback

Motivation was unanimously considered an important issue, although participants' views on what would motivate them differed.

For some participants the potential to improve movements through the training, or just completing the daily sessions, was intrinsically motivating.

> *The motivation is learning how to do something that you've forgotten how to do. I think that's motivation in itself [I3]*

> *Well every day it was quite an achievement really, for me, doing things on a daily basis once again [I4]*

Practicing more challenging actions, or a progression in the difficulty of actions might also provide a source of motivation:

> *…once you'd done those actions and you got confident you could do them very competently…to go on to something more challenging, different or challenging [I1]*

Some participants would find performance-related feedback helpful. It was also suggested that more feedback and encouragement could be built into the app:

> *It comes back to the old school days, if you're doing a thing, you like to know what progression you've made and if you're not doing it, it would be nice to find out what needs to be done to improve [I4]*

> *I'd like a bit more…words on the screen, telling you how you're doing and what to do next and then encouraging you, with the option of a voice-over, doing the same thing… Depending on which answer you tap, it could then say, well don't worry, keep going at it. Or, it could say, well done, that's good. (P3)*

Supplement S4: Difficulty ratings and motor imagery ratings during home training

(i)

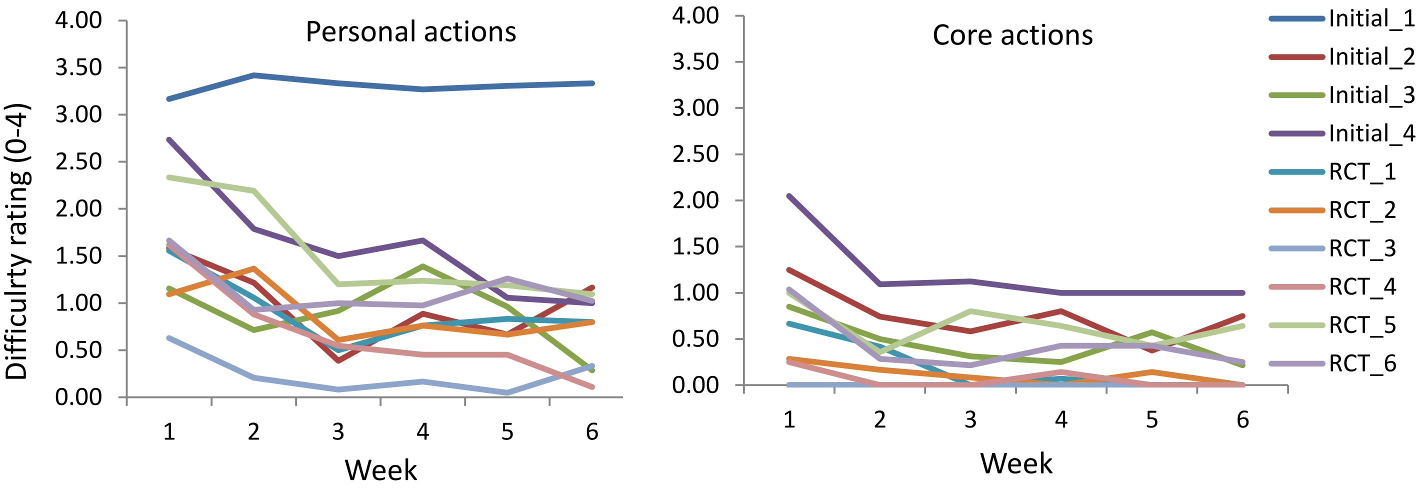

(ii)

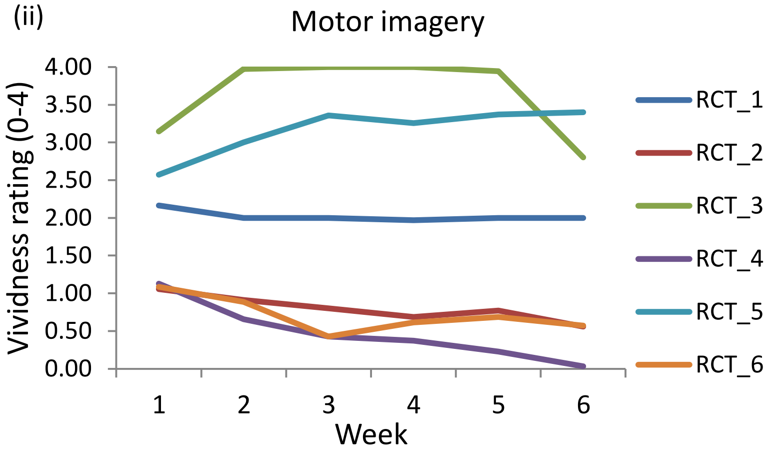

Figure S4. Average weekly ratings during the six-week intervention for (i) difficulty of personal and core actions in the initial testing and pilot RCT; (ii) motor imagery in the pilot RCT.

<u>Supplement S5: Exploratory outcome measures</u>

Performance on exploratory outcome measures in the initial testing and pilot RCT: median pre- and post- scores, change (baseline minus follow-up) and interquartile range of change score.

Group	DextQ-24	KVIQ	Simple RT (ms)	Choice RT (ms)	PDQ-39
Initial testing	36.5; 33.0<br>(2.0; 1.25)	107.5; 105.5<br>(-7.50; 27.6)	323; 296<br>(17.0; 42.0)	409; 388<br>(-14.13; 83.19)	22.50; 21.0<br>(-1.0; 6.75)
Pilot RCT:					
Intervention	36.5; 34.5<br>(2.5; 6.25)	122.0; 117.0<br>(4.50; 22.8)	309; 312<br>(-1.13; 34.13)	428; 395<br>(18.25; 129.44)	41.5; 36.0<br>(-1.50; 11.5)
Control	26.0; 26.0<br>(0.0; 3.0)	130.0; 118.0<br>(7.0; 30.8)	356; 349<br>(-6.75; 34.13)	395; 427<br>(-44.75; 52.13)	13.50; 7.0<br>(2.50; 5.0)

Note: Positive change values indicate improvement, except on the KVIQ.
